# Isolation and Identification of High Biomass and Lipid Productivity *Euglena* Strain from Tropical Malaysian Environments for Enhancement of Biofuel Production

**DOI:** 10.1007/s10126-025-10503-3

**Published:** 2025-08-23

**Authors:** Sabrina Aghazada, Kengo Suzuki, Yu Inaba, Kohei Atsuji, Koji Iwamoto

**Affiliations:** 1 Department of Chemical and Environmental Engineering, Malaysia Japan International Institute of Technology, Universiti Teknologi Malaysia, Jalan Sultan Yahya Petra, 54100 Kuala Lumpur, Malaysia; 2 Euglena Co., Ltd, 1-6, Suehiro-Cho, Tsurumi-Ku, Yokohama City, Kanagawa 230-0045 Japan; 3 RIKEN BZP, 1-7-22 Suehiro-Cho, Tsurumi-Ku, Yokohama City, Kanagawa 230-0045 Japan; 4 Euglena Malaysia Sdn. Bhd, Jalan Sultan Yahya Petra, 54100 Kuala Lumpur, Malaysia

**Keywords:** *Euglena*, Isolation, Identification, ITS2, Malaysia

## Abstract

The microalgae *Euglena* holds promise for biofuel production due to its high lipid content. However, the lipid productivity of current species/strains for biofuel production remains suboptimal due to limitations in strain selection. Therefore, this study aims to isolate and identify novel *Euglena* species or strains with high biomass and lipid productivity to enhance biofuel production from a desirable environment. Malaysia’s tropical climate with abundant sunlight and water resources provides an ideal environment for microalgae cultivation. Therefore, this research is conducted in Malaysia for effective utilization. Accordingly, water samples were collected from various Raja Musa Forest Reserve habitats in Selangor, Malaysia including peatland, paddy fields and the Kuala Selangor River. The samples were isolated using the single-cell pickup technique. The isolated samples were cultivated using the test tube system. The biomass and lipid productivity were quantified using the gravimetric technique. The top novel lipid-producing *Euglena* strain SAB-3 was identified through scanning light microscopy and phylogenetic analysis of the ITS2 region. The Sab-3 was placed within the *Euglena gracilis* clade through this analysis, showing close similarity to the *E. gracilis* SAG strain. SAB-3 dominated high biomass productivity (0.704 g L^1^ day^1^), high lipid productivity (0.051 g L^1^ day^1^) and relatively high specific growth rate (1.091 day^1^) with a shorter cultivation time of 7 days compared to the *E. gracilis* (NIES-48).

**Supplementary Information:**

The online version contains supplementary material available at 10.1007/s10126-025-10503-3.

## Introduction

Global energy demand continues to rise due to rapid population growth and industrialization. This demand is primarily met through fossil fuels, which contribute significantly to global warming and are major threat to the environment, economy and society (Chhandama et al. 2023; Ezhumalai et al. 2024; Merlo et al. 2021). As a result, increasing attention has been directed toward alternative and renewable energy sources. One of the renewable energy sources is microalgae biofuel, which can potentially replace fossil-based fuels (Singh et al. 2023). Microalgae biofuel an energy source derived from microalgae biomass (Arsalan and Iqbal. 2023). According to Chen et al. (2013) and Zhu et al. (2012) microalgae-based biofuel offers several advantages such as high photosynthesis efficiency, highly efficient carbon dioxide fixation and high bioproduct (e.g., pigments, carbohydrates, lipids, etc.) accumulation. Moreover, microalgae cultivation does not compete with food resources, arable land or freshwater supplies. Various microalgae species have been explored for biofuel production particularly green algae (*Acutodesmus, Botryococcus*, *Chlorella*, *Scenedesmus*), Eustigmatophyceae algae (*Nannochloropsis)* and euglenoids (*Euglena)* (Kim et al. 2023). Some microalgae species, e.g., *Chlorella*, have reached the techno-economic evaluation stage, achieving a biofuel production cost of approximately $0.5/L, while *Nannochloropsis* has reached a biofuel production cost of $2.3/L. However, large-scale commercialization remains elusive (Kim et al. 2023). In contrast, *Euglena* has been commercialized by Euglena Co. Ltd., Japan, which operates a refinery plant that produces biodiesel by mixing used cooking oils with wax ester from *Euglena gracilis.* Despite this advancement, production costs remain high (Suzuki. 2017; Kim et al. 2023).

*Euglena* is a unique genus of unicellular, flagellated microalgae capable of photosynthesis via the chloroplast. It belongs taxonomically to: *Euglena* (genus), Euglenaceae (family), Euglenales (order), Euglenoidea (class), Euglenozoa (phylum), Discoba (superphylum), Protista (kingdom), Eukaryote (domain) (Bicudo and Menezes. 2016; Borowitzka. 2018; Lei et al. 2024). It is characterized by a spindle-shaped body (15–500 µm in length) and green pigmentation. It lacks a cell wall and is surrounded by a cell membrane (pellicle) composed of proteinaceous strips, enabling cell movement (Borowitzka. 2018; He et al. 2021). Beyond its structural and metabolic features, *Euglena* is recognized for its nutritional and industrial potential. According to Euglena Co., this microalgae contains 59 types of nutrients, including vitamins (e.g., C and D), minerals (e.g., iron and calcium), proteins (e.g., lysine, alanine), carbohydrates (e.g., paramylon), pigments (e.g., chlorophyll, carotenoids) and lipids (e.g., DHA, EPA) (He et al. 2021; Inwongwan et al. 2019; Patil et al. 2024). Unlike other microalgae, *Euglena* exhibits plant-like and animal-like characteristics, enabling it to grow autotrophically, heterotrophically and mixotrophically (Borowitzka. 2018; Inwongwan et al. 2019). This metabolic flexibility gives it a distinct advantage in lipid accumulation under diverse environmental conditions. Its lipid content is moderate (20–30% DW) and lower than that of other oleaginous algae such as *Chlorella* and *Nannochloropsis* (Jung et al. 2021). Nevertheless, *Euglena* is uniquely capable of converting its storage carbohydrate (paramylon) into wax esters under anaerobic conditions, making it suitable for jet biofuel applications (Chen et al. 2022; Kim et al. 2023; Tsarenko et al. 2016). However, enhancing its lipid productivity for biofuel production remains challenging in terms of optimal species or strain selection (Kim et al. 2023). Therefore, this research aims to isolate and identify a novel *Euglena* strain from the Malaysian environment with high biomass and lipid productivity to enhance biofuel production.

## Materials and Methods

### Sample Collection

The water samples were collected from different sites at the Raja Musa Forest Reserve, Selangor, Malaysia including peatland, paddy fields and Kuala Selangor River (Sahabudin et al. 2022). The samples were transferred to the laboratory in 15 ml conical tubes and stored in a cool box. The primary and secondary cultures were then established under the conditions described below. The reference strain *E. gracilis* NIES-48 was obtained from the National Institute for Environmental Studies, Japan.

### Morphological Identification of Microalgae

Initial morphological identification of microalgae was performed using an inverted microscope (Eclipse TS100-F, Nikon, Japan) at 40 × magnification. Observations of cell size, shape and pigmentation were compared with descriptions from established databases (Telussa et al. 2022).

### Isolation of Microalgae

Isolation was conducted using the single-cell pickup technique described by Ota et al. (2019). Individual *Euglena* cells were sucked using a glass Pasteur pipette under an inverted microscope at 20× magnification. Each cell was washed 3 times with CM medium (Yahya et al. 2018) before being transferred to individual wells of a 96-well plate containing 200 µL CM medium. The cultures were maintained at 25 ± 1 °C under continuous illumination (100 µmol photons m^2^s^1^) for two weeks. Cultures were then sequentially inoculated every two weeks: first to 24-well plates containing 500 µL CM medium, then 6-well plates containing 2 mL CM medium and finally to the plastic cell culture flask containing 30 mL CM medium. The st﻿reak-plating method to remove bacterial contamination was employed following Bao et al. (2022). Agar plates were prepared with CM medium (pH 6) containing 0.5 µL/mL hexaconazole (5% w/w), 100 µg/mL ampicillin and 2% agar (Yahya et al. 2018). The medium was autoclaved at 121 °C for 20 minutes (Autoclave Sterilizer SX-700, Tomy, Japan). After cooling, the medium was poured into petri dishes and stored at 4 °C for 30 minutes. At the same time, cell samples were washed 3 times via centrifugation (Micro Centrifuge, MX-307, Tomy) at 1000 × g and 20 ℃ for 3 minutes. The washed cells were streaked onto agar plates using a sterile inoculating loop. Plates were wrapped with specific tape and incubated under continuous light of 100 µmol photons m^−2^ s^−1^ and 25 ± 1 °C. After one week, visible *Euglena* colonies appeared; individual colonies were picked up and transferred into a new inoculum plate containing fresh medium for further cultivation (Akinyemi and Olukunle 2022). The 4 most rapidly growing *Euglena* species or strains were selected from the isolates based on visible growth rate assessment for further investigation.

### Cultivation

Microalgae cultures were maintained using CM medium with pH 3.5 (Cramer and Myers. 1952) under 10% (v/v) CO₂ and continuous light at 100 µmol photons m^2^s^1^ at 29 °C. For experimental cultivation, triplicate cultures were grown in 100 mL glass test tubes containing 60 mL CM medium. The initial cell density was adjusted to 0.04 optical density (OD). Growth was monitored every 24 hours by measuring OD at the wavelength of 750 nm using a UV–Vis spectrophotometer (UV-1900, Shimadzu, Japan). The specific growth rate (day^-1^) was calculated during the exponential phase of growth using Eq. (1) (Sahabudin et al. 2022):

1 $$\mu=\frac{\mathrm{In}\;\left(\mathrm{OD}_\text2\right)-\mathrm{In}\boldsymbol\;\boldsymbol(\mathrm{OD}_\text1)}{ t_\text2-t_\text1}$$

where:

**μ:** specific growth rate (day⁻¹),

**OD**_**1**_**:** optical density at initial time t_1_,

**OD**_**2**_**:** optical density at late time t_2_,

**t**_**2**_**-t**_**1**_**:** time interval (days),

**In:** natural logarithm.

### Biomass Evaluation

At the stationary phase, 50 mL of each culture was harvested via centrifugation (ThermoFisher Scientific, USA) at 5000 × g for 5 minutes at 25 °C. The resulting pellet was stored at -20 °C until further processing. Frozen wet biomass was freeze-dried at -45 °C for 12 hours using a freeze-dryer (FDU-1110, Eyela, Japan). The dry weight of the cells was measured using an analytical balance (HR-250AZ, AND, Australia). Biomass content and productivity were calculated using Eqs. (2) and (3), respectively (Suyono et al. 2024):

2 $$Biomass Content \left(g{L}^{-1}\right)=\frac{\text{Final Weight of Sample}-\text{Intial Weight of Sample }}{\text{Sample Volume}}$$

3 $$Biomass Productivity \left(g {L}^{-1}da{y}^{-1} \right)=\frac{\text{Max Biomass}-\text{Intial Biomass }}{\text{Biomass Peak Time}-\text{Biomass Start Time}}$$

### Lipid Evaluation

Lipids were extracted from the dried biomass using the Folch method, which utilizes a 2:1 (v/v) chloroform: methanol mixture (Folch et al. 1957). The extracted lipid was dried by 2 hours using a spin dryer (VC-15 s, Taitec, Japan); the final lipid weight was determined gravimetrically using an analytical balance (HR-250AZ, AND, Japan) and the lipid content and lipid productivity were calculated using Eqs. (4) and (5), respectively (Suyono et al. 2024):

4 $$Lipid Content \left(g {L}^{-1}\right)=\frac{\text{Final Weight of Sample}-\text{Intial Weight of Sample }}{\text{Sample Volume}}$$

5 $$Lipid Productivity \left(g{L}^{-1}da{y}^{-1}\right)\frac{\text{Max Lipid}-\text{Intial Lipid}}{\text{Lipid Peak Time}-\text{Lipid Start Time}}$$

### Molecular Identification of Microalgae

The top lipid-producing *Euglena* strain (SAB-3) was identified through phylogenetic analysis targeting the Internal Transcribed Spacer 2 (ITS2) region. A 1.5 mL aliquot of the stationary phase culture was harvested via centrifugation at 5000 × g for 5 minutes (MX-307, Tomy, Japan). The total plastid genomic DNA was extracted using the NucleoSpin® Tissue DNA extraction kit according to the manufacturer's protocol instructions (Takara Bio Inc., Shiga, Japan). The quality of DNA was assessed using a Nanodrop Spectrophotometer (NanoDrop™ 2000/2000c, Thermo Scientific, USA). The Polymerase Chain Reaction (PCR) amplification of the ITS2 region was conducted to confirm the strain-level identity (Tarannum et al. 2020). The reactions were performed using a thermal cycler (Bio-Rad, USA) with the following primers: ITS2-Forward 2 (5'TTCTGAGGAAGGACACAGCAGC3'), ITS2-Reverse (5'TTCCTCCACTGAGTGATATGC3') (Eurofins Scientific, Yokohama, Japan) and in combination with MY PCR Kit 1 (Vivantis, Selangor, Malaysia). The PCR mixture (25 µL total volume) included: 12.5 µL 2 × Taq Master Mix, 1 µL each of forward and reverse primers (conc.: 0.5 µM), 2 µL DNA template (conc.: 0.02–0.09 µg/µL) and 8.5 µL nuclease-free water. This study employed the 3-step PCR cycling protocol consisting of 35 cycles under the following conditions (Hotos et al. 2023): initial denaturation at 94 °C for 2 minutes (final denaturation at 94 ℃ for 30 seconds), annealing at 52 °C for 30 seconds, extension at 72 °C for 30 seconds (final extension at 72 °C for 7 minutes) and hold at 4 °C indefinitely. Notably, the annealing temperature was selected based on the primer's guidelines. Gel electrophoresis confirmed the PCR products using 2% agarose gel prepared with MY PCR Kit 1 (Vivantis, Selangor, Malaysia) and a 1 kb DNA ladder (10,000 bp) using Mupid-2Plus (Takara Bio, Japan) and Heating Block (Daihan-Sci, Korea). The gel was visualized using the Quantum GelDoc (Vilber, USA) and VisionCapt software. The Sanger sequencing was conducted using the same primers by Wan Care Scientific Sdn. Bhd (Kuala Lumpur, Malaysia). The sequence alignment was performed using GeneStudio v1.41. The homology comparison of the product was carried out using the strain data from the SAG Culture Collection (Sammlung von Algenkulturen), University of Göttingen, Germany. The phylogenetic tree was established using MEGA X version 11 software.

### Statistical Analysis

The One-way ANOVA and *t*-tests were conducted using Microsoft Excel to determine statistically significant differences between experimental groups.

## Results and Discussion

### Sample Collection

**Fig. 1:**
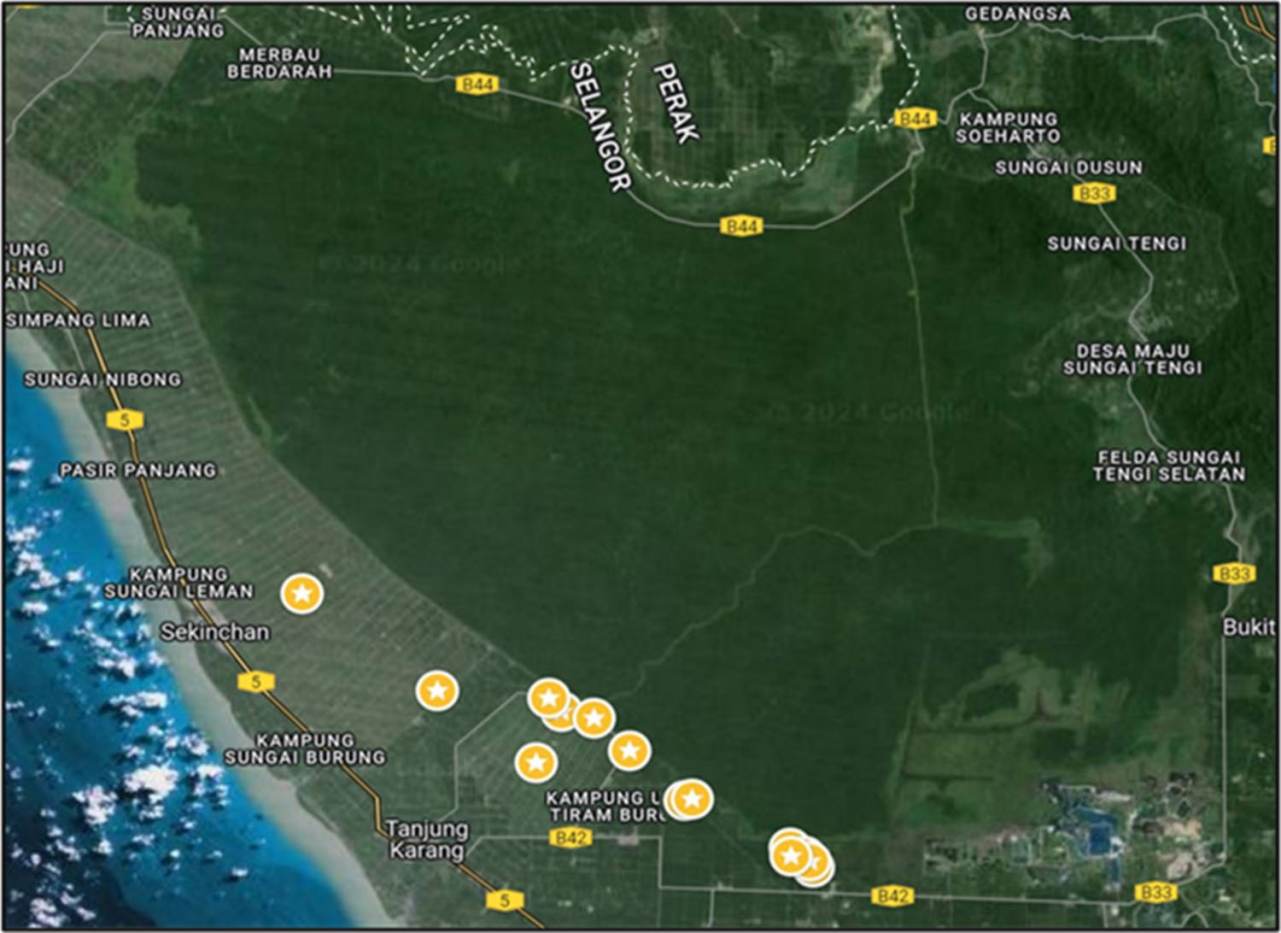
Map of the sampling sites in the Raja Musa Forest Reserve. Stars indicate the locations where samples were collected. Base map source: © Google Maps; image modified by the author

In total, 27 water samples were collected from various sites of the Raja Musa Forest Reserve: 16 samples from peatlands, 3 from Kuala Selangor River and 8 from paddy field (Fig. 1). At each sampling site, the environmental parameters such as pH and temperature varied significantly, with pH ranging from 3.3 to 11.6 and temperature ranging from 26 °C to 40 °C. The Supplementary Information (Table S1) provides detailed data on sampling conditions.

### Morphological Identification of Microalgae

**Fig. 2:**
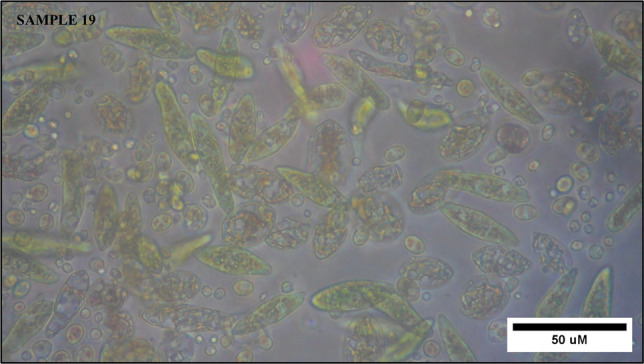
Morphological image of sample 19 (Mixture) collected from the Kuala Selangor River, showing *Euglena* under an inverted microscope at 40× magnification with a black scale bar of 50 µm generated by ImageJ

The microscopic examination at 40× magnification confirmed the presence of *Euglena* sp. in sample 19, which was collected from the Kuala Selangor River (3°28′26.3"N, 101°14′05.5"E) (Fig. 2). The observed cells exhibited morphological characteristics of *Euglena*, including a greenish coloration, spindle-shaped bodies, active motility and lengths generally range from about 35 to 55 µm. These features are consistent with previously reported descriptions (Chen et al. 2022; Ruiz et al. 2004). At the time of collection, the water displayed a pH 5.5 and a temperature of 33 °C, favorable conditions for *Euglena* growth (Kitaya et al. 2005; Mujahidah et al. 2024). The elevated temperature further suggests that microalgae were present near the water surface, likely engaging in active photosynthesis.

### Isolation of Microalgae

**Fig. 3:**
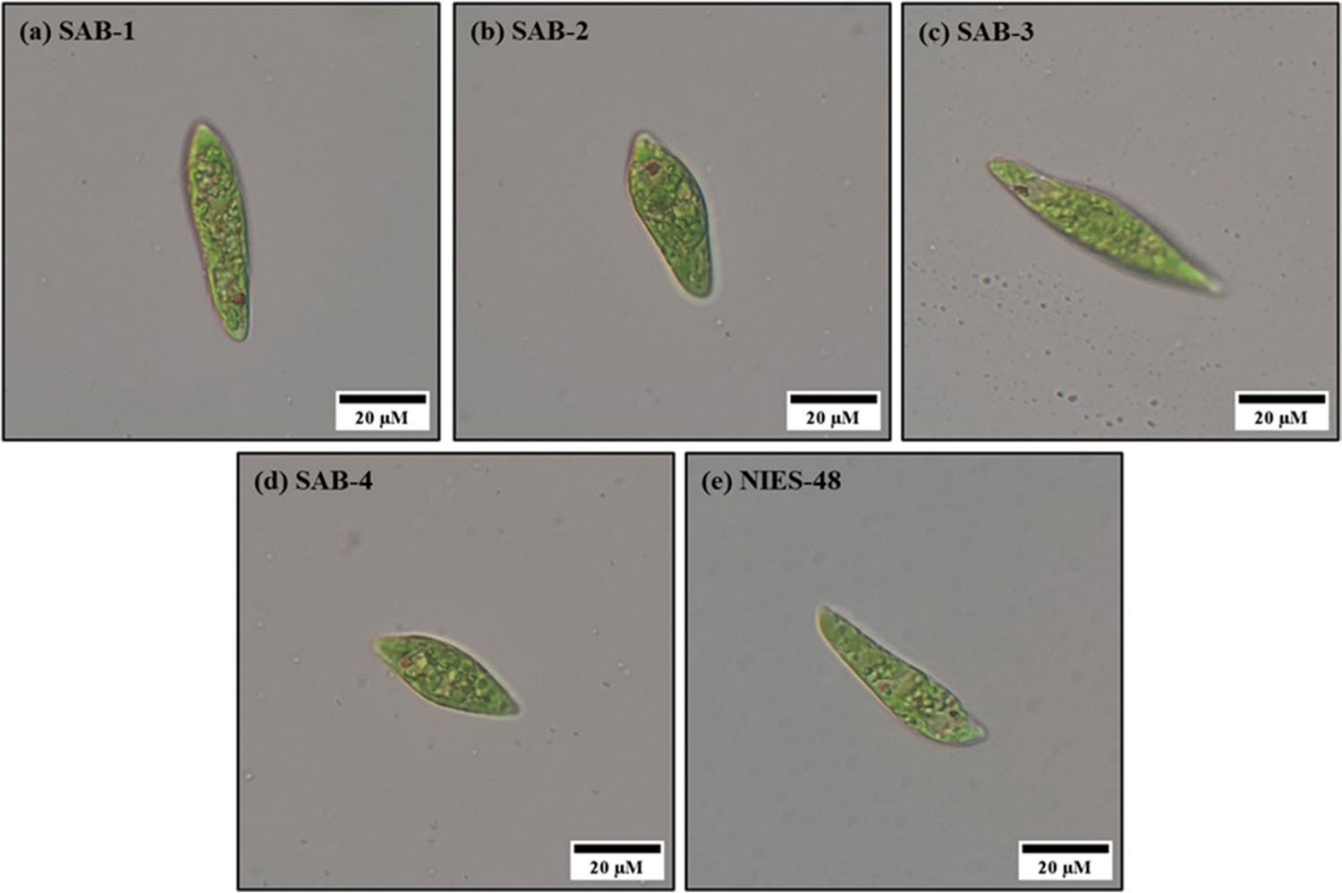
Microscopic images of isolated strains: (**a**) SAB-1, (**b**) SAB-2, (**c**) SAB-3, (**d**) SAB-4 and reference strain: (**e**) NIES-48, with cell lengths ranging from 35 to 55 µm. Black scale bars: 20 µm

In total, 24 isolates were successfully obtained from the mixture culture sample 19 using the single-cell pickup technique. Among these, the four fastest-growing isolates were selected for further analysis and designated SAB-1, SAB-2, SAB-3 and SAB-4 (Fig. 3). The isolates were successfully separated from bacterial contamination via the streak plating technique on agar plates containing CM medium, supplemented with hexaconazole (fungicide) and ampicillin. The plates containing algal cells were examined every two days using an inverted microscope at 20× and 40× magnifications. Distinct single colonies were observed on day 8. The SAB isolates were cultured in CM medium and grown at 25 ± 1 °C under continuous light at 100 µmol photons m⁻^2^ s⁻^1^ for the subsequent studies.

### Cultivation, Biomass and Lipid Evaluation

**Fig. 4:**
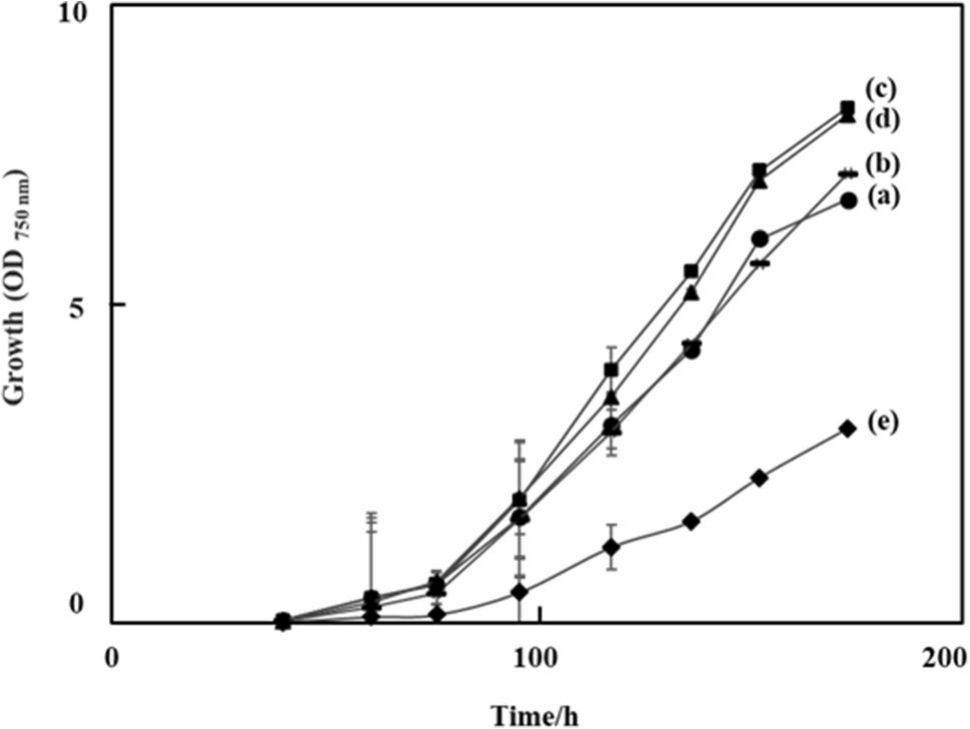
Growth curves of newly isolated SAB strains: (**a**) SAB-1, (**b**) SAB-2, (**c**) SAB-3, (**d**) SAB-4 and reference strain: (**e**) NIES-48 during 7 days of cultivation in test tube system under continuous light at 100 µmol photons m⁻^2^ s⁻^1^, 29 °C, 10% CO₂ and measured at OD_750 nm_

**Fig. 5:**
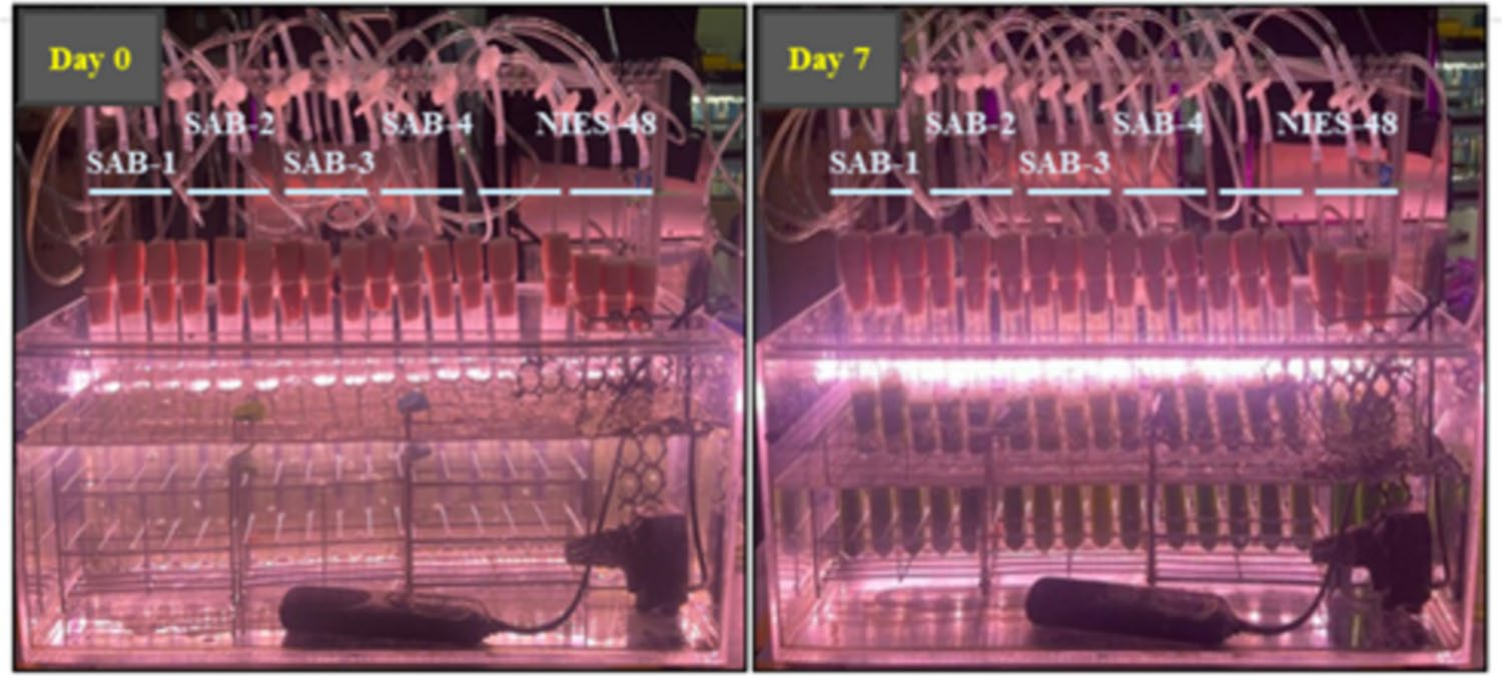
Visual comparison of SAB strains and reference strain NIES-48 on day 0 and day 7 of cultivation

**Fig. 6:**
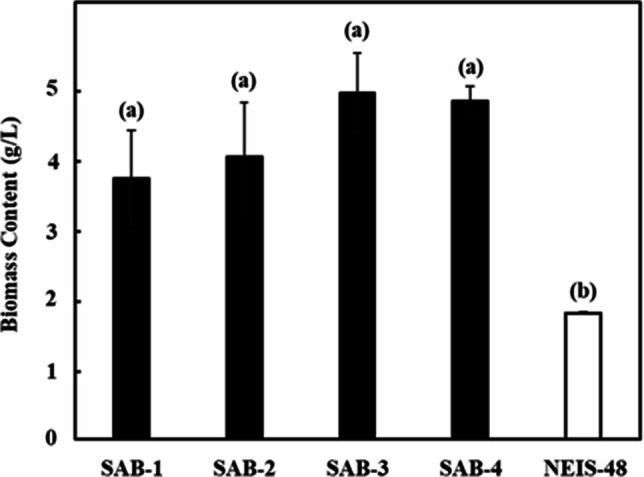
Biomass content of SAB strains and NIES-48 strain on day 7 of cultivation in CM medium (stationary phase). A P ≤ 0.05 value indicates a significant difference between group (**a**) SAB strains and group (**b**) NIES-48 strain

**Fig. 7:**
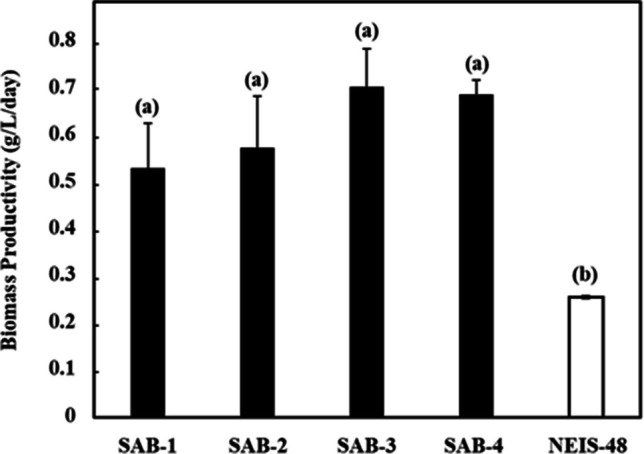
Biomass productivity of SAB strains and NIES-48 strain on 7 days of cultivation. A P ≤ 0.05 value specifies a significant difference between group (**a**) SAB strains and group (**b**) NIES-48 strain

**Fig. 8:**
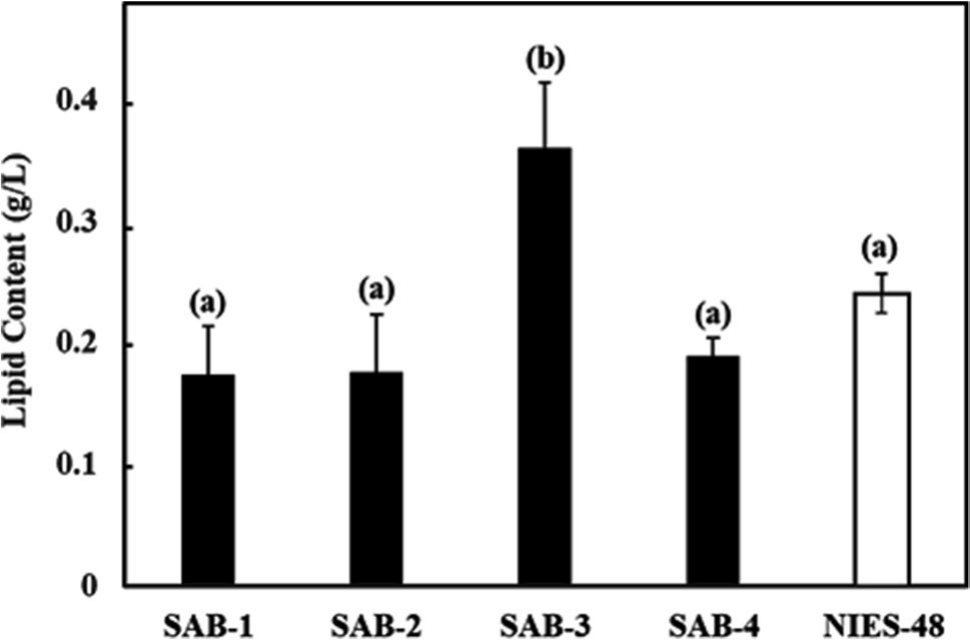
Lipid content of SAB strains and NIES-48 standard strain after 7 days of cultivation. A P ≤ 0.05 value offers a significant difference between the group (**a**) SAB-1, SAB-2, SAB-4 and NIES-48 strains and group (**b**) SAB-3 strain

**Fig. 9:**
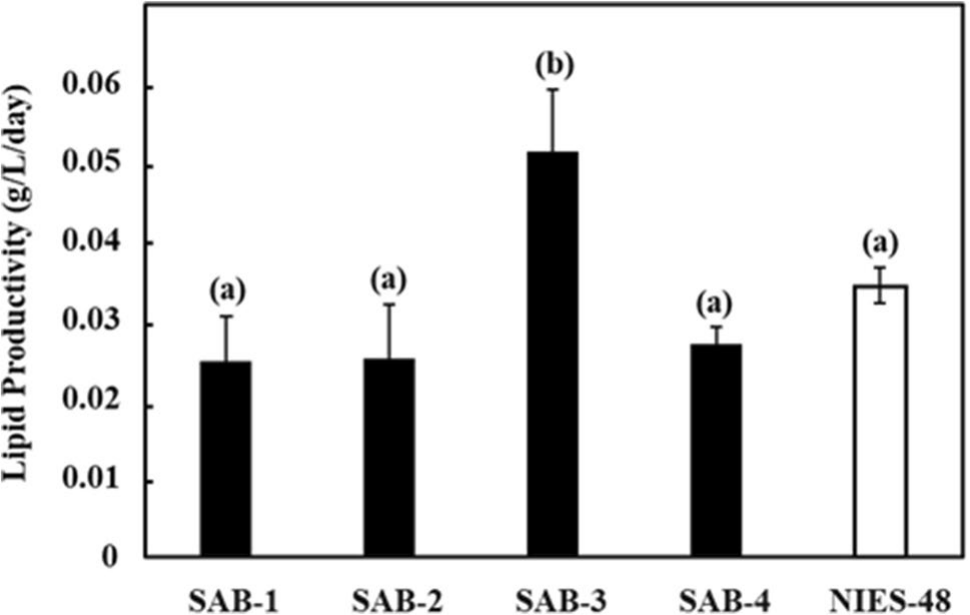
Lipid productivity of SAB strains and NIES-48 strain after day 7 of cultivation. A P ≤ 0.05 value presents a significant difference between the group (**a**) SAB-1, SAB-2, SAB-4 and NIES-48 strains and group (**b**) SAB-3 strain

The growth profiles of the isolated SAB strains were monitored spectrophotometrically over the 7-day cultivation period, as presented in Fig. 4. Figure 5 shows the visual culture observations on days 0 and 7. The growth curves for all strains exhibited a characteristic pattern: a lag phase (days 0-2) during which cells adapted to the environment; an exponential phase (days 2-5) and a late exponential phase (days 5-6) marked by rapid cell growth; a stationary phase (days 6-7) where growth slowed significantly. A caloric restriction phase was observed after day 7, indicating a biological lifespan of approximately 7 days under the given conditions. The specific growth rates (µ) were calculated during the exponential phase (days 1–3). The SAB-4 strains exhibited the highest specific growth rate (1.221 day⁻^1^), while the reference strain NIES-48 showed the lowest (0.391 day⁻^1^). The specific growth rates of SAB-2, SAB-3 and SAB-1 were 1.155 day⁻^1^, 1.091 day⁻^1^ and 0.939 day⁻^1^, respectively. The final OD_750__nm_ of the new SAB strains on day 7 was higher than the NIES-48 strain, which also indicates higher biomass accumulation. The gravimetric analysis was used to assess the dry biomass of the newly isolated strains compared to the standard NIES-48 strain. The biomass content and biomass productivity at the stationary phase (day 7) are shown in Figs. 6 and 7, respectively. Among all isolates, SAB-3 presented the highest values across all biomass and lipid parameters, including biomass content (4.930 g L^−1^), biomass productivity (0.704 g L^−1^ day^−1^), lipid content (0.359 g L^−1^) and lipid productivity (0.051 g L^−1^ day^−1^). Although its specific growth rate (1.091 day^−1^) was slightly lower than SAB-4 and SAB-2, it remained substantially higher than strain SAB-1 (0.939 day^−1^) and NIES-48 (0.391 day^−1^). Notably﻿, SA﻿B-3 strain's biomass productivity was 2.75 times higher and its lipid productivity 1.5 times higher than the NIES-48 strain, emphasizing its superior potential for biofuel applications. In contrast, the NIES-48 strain showed the lowest biomass content (1.793 g L^−1^) and biomass productivity (0.256 g L^−1^ day^−1^). It exhibited a relatively higher lipid content (0.240 g L^−1^) and lipid productivity (0.034 g L^−1^ day^−1^) than SAB-1, SAB-2 and SAB-4, but not SAB-3. SAB-4 demonstrated the highest specific growth rate (1.221 day^−1^) and high biomass content (4.809 g L^−1^day^−1^), suggesting potential for efficient biomass production. However, its lipid content (0.188 g L^−1^ day^−1^), was significantly lower, reducing its utility for lipid-based biofuel production. *t*-test results indicated a statistically significant difference in biomass content and productivity between group (a): SAB strains and group (b): NIES-48 strain P ≤ 0.05, as shown in Figs. 6 and 7. Further statistical analysis revealed a significant lipid content and productivity difference between SAB-3 and all other strains (SAB-1, SAB-2, SAB-4, and NIES-48), as presented in Figs. 8 and 9. Table 1 summarizes all performance indicators, including specific growth rate, biomass content, biomass productivity, lipid content and lipid productivity.

**Table 1:** Comprehensive comparison of specific growth rate, biomass content, biomass productivity, lipid content and lipid productivity of SAB strains and the standard strain NIES-48

Strain Name	Specific Growth Rate (day^−1^)	Biomass Content (g L^−1^)	Biomass Productivity (g L^−1^ day^−1^)	Lipid Content (g L^−1^)	Lipid Productivity (g L^−1^ day^−1^)
SAB-1	0.939	3.716	0.530	0.173	0.024
SAB-2	1.155	4.025	0.575	0.175	0.025
SAB-3	1.091	4.930	0.704	0.359	0.051
SAB-4	1.221	4.809	0.687	0.188	0.026
NIES-48	0.391	1.793	0.256	0.240	0.034

Compared to *E. gracilis* 815, a recently described high-performance *Euglena* strain (Chen et al. 2022), SAB-3 demonstrated 6.1 times higher biomass and 1.5 times higher lipid content. While the lipid concentration of SAB-3 (7.3%) was slightly lower than reported for *E. gracilis* 815, this may not be a limiting factor. As noted in a previous study, *Euglena* can convert stored paramylon into wax esters under anaerobic and dark conditions, suitable for biofuel applications (Kim et al. 2023). The sequencing results further validated that the fragment was 711 bp long, indicating high sequence quality suitable for molecular analysis. For phylogenetic analysis, multiple sequence alignment was performed using ClustalW, followed by constructing a phylogenetic tree via the neighbor-joining method with 1000 bootstrap replicates. The resulting phylogenetic tree placed strain SAB-3 within the *E. gracilis* clade, closely grouped with the *E. gracilis* SAG strain, as illustrated in Fig. 11. The ITS2 region is widely regarded as a reliable molecular marker for identifying and classifying microalgae due to its balance of interspecies variability and intraspecies conservation, consistent with findings in other taxa such as *Selenastrum densum* (Liu et al. 2024a, b). The phylogenetic positioning of SAB-3 and its growth preference for acidic conditions, a known characteristic of *E. gracilis* (Kitaya et al. 2005), supports its classification within this species. This molecular approach enhances taxonomic resolution and confirms the identity of SAB-3, reinforcing the utility of ITS2-based phylogenetic analysis in microalgae systematics.

### Molecular Identification of Microalgae

**Fig. 10:**
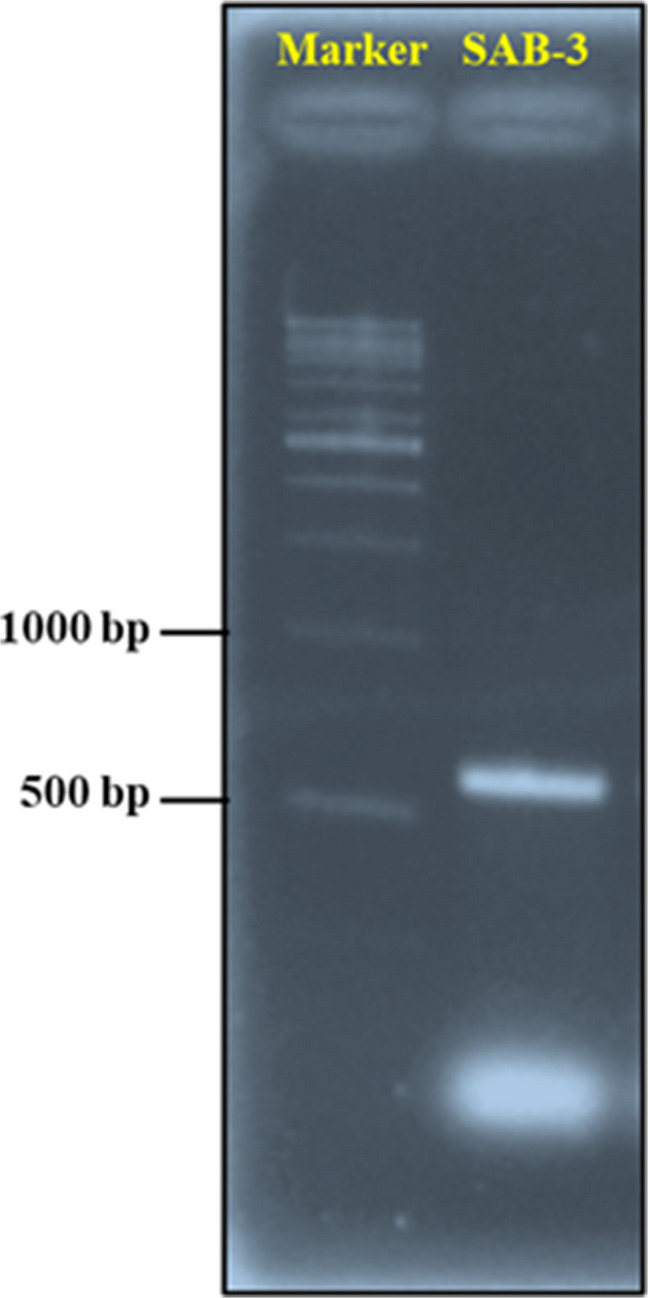
Electropherogram of the ITS2 PCR product from strain SAB-3, showing DNA fragment size between 500 and 1000 bp

**Fig. 11:**
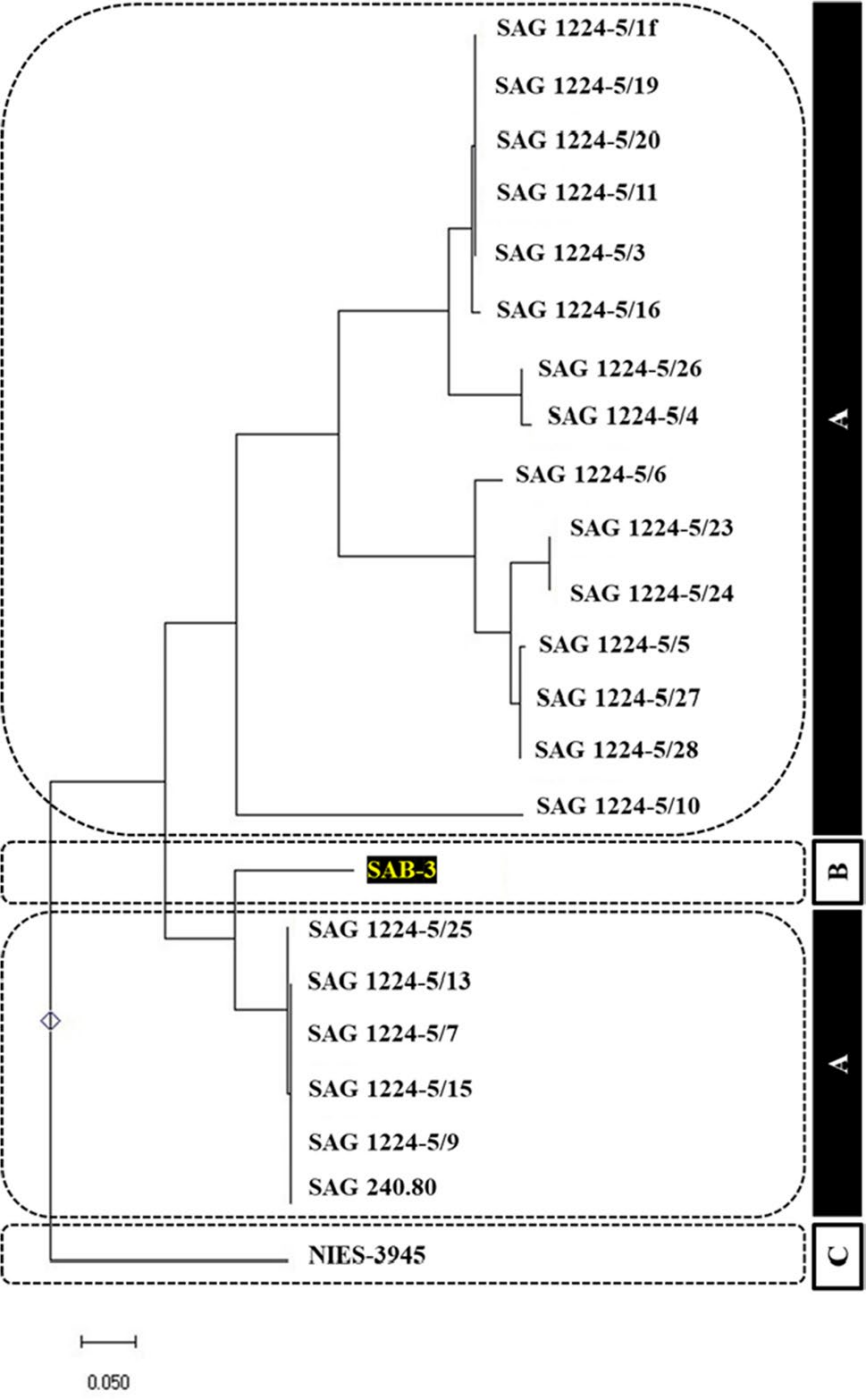
Phylogenetic tree constructed using the neighbor-joining method (1000 bootstrap replicates) based on ITS2 sequences. Includes: (**A**) 21 type strains of *E. gracilis* from the SAG Culture Collection (Sammlung von Algenkulturen), University of Göttingen, Germany; (**B**) Isolated strain SAB-3 and (**C**) Outgroup: *E. gracilis* NIES-3945 strain

The top lipid-producing *Euglena* strain (SAB-3) genomic DNA was successfully extracted and evaluated for quality and yield. The spectrophotometric analysis revealed an A₂₆₀/A₂₈₀ ratio of 2.06, indicating high purity. The DNA concentration was measured at 0.062 µg/µL, sufficient for downstream applications, including PCR amplification and sequencing. The ITS2 region was successfully amplified via PCR. The gel electrophoresis confirmed that the amplified fragment was between 500 and 1000 base pairs (bp), as shown in Fig. 10. The sequencing results further validated that the fragment was 711 bp long, indicating high sequence quality suitable for molecular analysis. For phylogenetic analysis, multiple sequence alignment was performed using ClustalW, followed by constructing a phylogenetic tree via the neighbor-joining method with 1000 bootstrap replicates. The resulting phylogenetic tree placed strain SAB-3 within the *E. gracilis *clade, closely grouped with the *E. gracilis* SAG strain, as illustrated in Fig. 11. The ITS2 region is widely regarded as a reliable molecular marker for identifying and classifying microalgae due to its balance of interspecies variability and intraspecies conservation, consistent with findings in other taxa such as *Selenastrum densum* (Liu et al. 2024). The phylogenetic positioning of SAB-3 and its growth preference for acidic conditions, a known characteristic of E. gracilis (Kitaya et al. 2005), supports its classification within this species. This molecular approach enhances taxonomic resolution and confirms the identity of SAB-3, reinforcing the utility of ITS2-based phylogenetic analysis in microalgae systematics.

## Conclusion

This study successfully isolated and identified a novel high lipid-producing *Euglena* strain SAB-3 from the Malaysian environment. Molecular identification through the ITS2 region confirmed its classification within the *E. gracilis* clade, showing close similarity to the *E. gracilis* SAG strain. This novel strain demonstrated high biomass productivity (0.704 g L^−1^ day^−1^), lipid productivity (0.051 g L^−1^ day^−1^) and a relatively high specific growth rate (1.091 day^−1^) with a shorter lifetime of 7 days compared to the *E. gracilis* NIES-48 strain. This finding contributes to the enhancement of biofuel potential.

## Supplementary Information

Below is the link to the electronic supplementary material.

Supplementary file1 (DOCX 19 KB)

## Data Availability

The original contributions presented in this study are included in the article and Supplementary Material. Further inquiries can be directed to the corresponding author.
