# Correction to: Isolation and Identification of High Biomass and Lipid Productivity *Euglena* Strain from Tropical Malaysian Environments for Enhancement of Biofuel Production

**DOI:** 10.1007/s10126-026-10574-w

**Published:** 2026-03-05

**Authors:** Sabrina Aghazada, Kengo Suzuki, Yu Inaba, Kohei Atsuji, Koji Iwamoto

**Affiliations:** 1 Malaysia Japan International Institute of Technology, Universiti Teknologi Malaysia, Jalan Sultan Yahya Petra, 54100 Kuala Lumpur Malaysia; 2 Euglena Co., Ltd, 1-6, Suehiro-Cho, Tsurumi-Ku, Yokohama City, Kanagawa 230-0045 Japan; 3 RIKEN BZP, 1-7-22 Suehiro-Cho, Tsurumi-Ku, Yokohama City, Kanagawa 230-0045 Japan; 4 Euglena Malaysia Sdn. Bhd, Jalan Sultan Yahya Petra, Kuala Lumpur, 54100 Malaysia

**Correction to: Marine Biotechnology (2025) 27:127**

**https://doi.org/10.1007/s10126-025-10503-3**

In this article, the following corrections have been made:

1. Department in affiliation 1 has been added, the correct affiliation 1 is now “Department of Chemical and Environmental Engineering, Malaysia Japan International Institute of Technology, Universiti Teknologi Malaysia, Jalan Sultan Yahya Petra, 54100 Kuala Lumpur, Malaysia”.
2. Biomass Evaluation, Molecular Identification of Microalgae and Statistical Analysis should be heading 2 (Materials and Methods). As well as u, OD1, OD2, t2-t1 should be the same size as the text, but just bold. “µ: specific growth rate per day,” has been replaced with “µ: specific growth rate (day⁻¹),”.
3. Space should be inserted on “of100” (Materials and Methods, Isolation of Microalgae, line 26).
4. After final extension at 72 °C for 7 min, a bracket should be added (Materials and Methods, Molecular Identification of Microalgae, line 28). Hyphen should also added between 1 and 3 (days 13) (Result and discussion, Cultivation Biomass and Lipid evaluation, line 13). Remove one hyphen from this (0.051 g L−-1 day − 1) (line 29).
5. On page 8, the second and third paragraphs should be combined.
6. Author’s contribution, the first line “SA conceptualized the research idea, performed the experiments and wrote both the original draft and final version” should be corrected to “SA conceptualized the research idea, designed the methodology, carried out the investigation, performed the experiments and wrote both the original draft and the final version”.
7. Data Availability statement has been updated to “The original contributions presented in this study are included in the article and Supplementary Material. Further inquiries can be directed to the corresponding author.”
8. Figures 2-4,6-11 have been revised to improved their quality; also Figs. 2 and 3, the scale bar has changed.
9. In Funding statement, "The" has been added to make "The open access funding provided by the Ministry of Higher Education Malaysia and Universiti Teknologi Malaysia. This work was supported by the Euglena Co. Ltd., Japan (Grant No. 4B889)." instead of "Open access funding provided by the Ministry of Higher Education Malaysia and Universiti Teknologi Malaysia. This work was supported by the Euglena Co. Ltd., Japan (Grant No. 4B889)."

The original article has been corrected.

