# Supplementary material for: Isolation and Identification of High Biomass and Lipid Productivity Euglena Strain from Tropical Malaysian Environments for Enhancement of Biofuel Production
Source: Mar Biotechnol (NY). 2025 Aug 23;27(5):127. doi: 10.1007/s10126-025-10503-3 (PMC12374904; doi:10.1007/s10126-025-10503-3)
Supplement: Supplementary file 1 — Supplementary file1 (DOCX 19 KB) [file 10126_2025_10503_MOESM1_ESM.docx]

Table S1 Environmental and geographical details of the sample collection sites

| **Sample**  **No.** | **Geographical**  **Coordinate** | **pH** | **Temp.** | **Collection**  **Site** |
| --- | --- | --- | --- | --- |
| **1** | 3°25'07.0"N 101°18'39.4"E | 3.6 | 26 ℃ | **Peatland** |
| **2** | 3°25'07.0"N 101°18'39.4"E | 3.8 | 26 ℃ |  |
| **3** | 3°25'07.0"N 101°18'39.4"E | 3.8 | 26 ℃ |  |
| **4** | 3°25'15.1"N 101°18'35.2"E | 3.6 | 30 ℃ |  |
| **5** | 3°25'22.5"N 101°18'28.2"E | 3.4 | 28 ℃ |  |
| **6** | 3°25'22.5"N 101°18'28.2"E | 3.7 | 26 ℃ |  |
| **7** | 3°25'22.2"N 101°18'11.5"E | 3.7 | 28 ℃ |  |
| **8** | 3°25'33.0"N 101°18'10.3"E | 3.9 | 26 ℃ |  |
| **9** | 3°25'33.0"N 101°18'10.3"E | 3.9 | 26 ℃ |  |
| **10** | 3°26'36.7"N 101°15'58.7"E | 7.5 | 29 ℃ |  |
| **11** | 3°26'36.7"N 101°15'58.7"E | 7.2 | 31 ℃ |  |
| **12** | 3°26'38.4"N 101°16'08.5"E | 3.8 | 31 ℃ |  |
| **13** | 3°26'38.4"N 101°16'08.5"E | 3.5 | 31 ℃ |  |
| **14** | 3°27'27.1"N 101°12'53.1"E | 3.3 | 28 ℃ |  |
| **15** | 3°27'42.5"N 101°14'50.2"E | 6.2 | 32 ℃ |  |
| **16** | 3°27'42.5"N 101°14'50.2"E | 6.4 | 30 ℃ |  |
| **17** | 3°27'42.5"N 101°14'50.2"E | 4.4 | 34 ℃ | **Kuala Selangor Rever** |
| **18** | 3°28'26.3"N 101°14'05.5"E | 5.5 | 34 ℃ |  |
| **19** | 3°28'26.3"N 101°14'05.5"E | 5.5 | 34 ℃ |  |
| **20** | 3°28'34.2"N 101°13'25.2"E | 11.6 | 37 ℃ | **Paddy Field** |
| **21** | 3°28'34.2"N 101°13'25.2"E | 11.6 | 36 ℃ |  |
| **22** | 3°28'52.1"N 101°13'08.6"E | 7.4 | 33 ℃ |  |
| **23** | 3°29'02.8"N 101°10'50.1"E | 7.3 | 32 ℃ |  |
| **24** | 3°29'02.8"N 101°10'50.1"E | 7.2 | 30 ℃ |  |
| **25** | 3°29'02.8"N 101°10'50.1"E | 7.7 | 34 ℃ |  |
| **26** | 3°31'12.0"N 101°08'00.4"E | 7.8 | 40 ℃ |  |
| **27** | 3°31'12.0"N 101°08'00.4"E | 7.2 | 32 ℃ |  |
